# The Role of Mannose-Binding Lectin in Severe Sepsis and Septic Shock

**DOI:** 10.1155/2013/625803

**Published:** 2013-10-02

**Authors:** Gennaro De Pascale, Salvatore Lucio Cutuli, Mariano Alberto Pennisi, Massimo Antonelli

**Affiliations:** ^1^Department of Anesthesiology and Intensive Care, Catholic University of the Sacred Heart, Agostino Gemelli Hospital, 00168 Rome, Italy; ^2^Policlinico Universitario A. Gemelli, Università Cattolica del Sacro Cuore, Largo A. Gemelli 8, 00168 Rome, Italy

## Abstract

Severe sepsis and septic shock are a primary cause of death in patients in intensive care unit (ICU). Investigations upon genetic susceptibility profile to systemic complications during severe infections are a field of increasing scientific interest. Particularly when adaptive immune system is compromised or immature, innate immunity plays a key role in the immediate defense against invasive pathogens. Mannose-binding lectin (MBL) is a serum protein that recognizes a wide range of pathogenic microorganisms and activates complement cascade via the antibody-independent pathway. More than 30% of humans harbor mutations in MBL gene (MBL2) resulting in reduced plasmatic levels and activity. Increased risk of infection acquisition has been largely documented in MBL-deficient patients, but the real impact of this form of innate immunosuppression upon clinical outcome is not clear. In critically ill patients higher incidence and worse prognosis of severe sepsis/septic shock appear to be associated with low-producers haplotypes. However an excess of MBL activation might be also harmful due to the possibility of an unbalanced proinflammatory response and an additional host injury. Strategies of replacement therapies in critically ill patients with severe infections are under investigation but still far to be applied in clinical practice.

## 1. Introduction

Despite the diffusion of effective care bundles and the implementation of new technologies able to support organ function, severe sepsis and septic shock still represent a leading cause of intensive care unit (ICU) admission with a case fatality rate of 30–40% [1, 2]. Systemic inflammation, surrounding multiorgan failure and septic shock, results from a maladaptive unbalance between early antimicrobial immune reactions and uncontrolled local infection and inflammation. Innate immune system is the primitive first-line organism's response to invasive pathogens, and it interacts with other homeostatic patterns, including inflammation and coagulation. Early activation of immune response is mediated by soluble pattern recognition molecules that, in addition to complement proteins, cytokines, and coagulation factors, activate humoral and cellular effectors, identifying and neutralizing the invasive pathogen [3]. Mannose-binding lectin (MBL) is a soluble pattern recognition molecule which activates the lectin pathway of the complement system and the subsequent inflammatory mechanisms [4, 5]. Low MBL plasmatic levels, mainly due to genetic influences, have been largely described to be associated with susceptibility to invasive infections and poor outcome [6]. On the other hand, the excessive activation of this ancient protective system may be responsible for a detrimental unbalanced inflammatory and coagulation response, as observed in inflammatory diseases, transplant rejections, and diabetic nephropathy [7]. Many authors have investigated whether MBL may influence the susceptibility to common pathogens and the development of severe infections, but, still, there is no consensus about the clinical relevance of its deficiency or the indications for replacement therapies.

The purpose of this review is to summarize the results of relevant recent studies where the role of MBL in severe sepsis and septic shock has been investigated.

## 2. The MBL Protein and Its Deficiency

**Figure 1:**
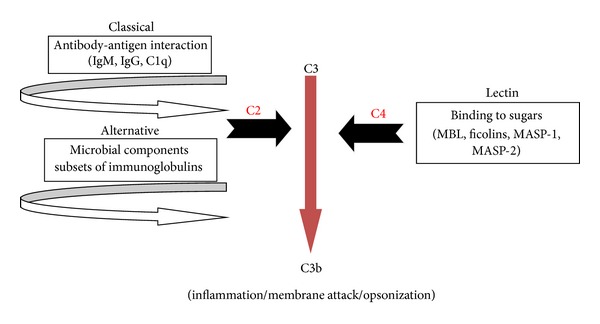
Complement activation pathways.

Mannose-binding lectin is a serum calcium-dependent protein, synthesized by the liver and is detectable in the sites of inflammation, particularly in epithelial-lining fluid [5]. Small amounts of this protein are also produced in other organs (kidney, thymus, tonsil, small intestine, and vagina). MBL is a collectin (collagen-like lectin) and is characterized by a high-ordered oligomeric structure that is essential for its function and interaction with MBL-associated serine proteases (MASPs) [8, 9]. MBL harbors a carbohydrate recognition domain (CRD) through which it binds to specific carbohydrates (i.e., mannose or N-acetylglucosamine) exposed on pathogenic agents surface, and it is therefore called a “pattern-recognition molecule” [4]. Subsequently MASPs (mainly MASP-2) are able to trigger the lectin complement pathway cleaving C4 and C2 to form C3 convertase. Complement system may be activated by three pathways: the classic and the alternative ones are antibody dependent and belong to the adaptive immune response; the lectin one is antibody independent and, as part of the innate immune system, comes into play within the first 12 hours from microorganisms' contact [10] (Figure 1). MBL plays a central role as a first-line defense against invading pathogens by triggering complement system, directly mediating opsonophagocytosis, and possibly functioning as a toll-like receptor coreceptor [11]. In humans there are two genes that might code for MBL, but only MBL2 gene is functional, located on the long arm of chromosome 10 [12]. MBL deficiency may be due to the presence of single nucleotide polymorphisms (SNP) either in the gene-coding or in the promoter regions. The wild-type gene is called “A” (homozygous haplotype, A/A); instead the variants alleles are widely classified as “0” (0/A and 0/0). Three points mutations involve the exon 1, identifying allele B (Codon 54), C (Codon 57), and D (Codon 52). Additionally, many SNPs may affect the promoter region and determine low MBL protein serum levels and activity (variants H/L, Y/X, and P/Q). Even though all these mutations could be combined with exon 1 alleles, seven haplotypes are typically found in humans genome [13, 14]. 

These polymorphisms determine the production of unstable proteins, with shorter half-life, mainly due to the absence of the high-ordered oligomeric structure. These variants are not able to efficiently activate the complement pathway [15].

Healthy individuals (genotype A/A) generally present MBL levels above 1000 ng/mL. In the newborn this protein is detectable at concentrations of two-thirds of their mothers. Normal levels are reached within a month [16]. MBL levels are not influenced by age, circadian cycle, and physical exercise and, during inflammation, do not increase over 3-4 folds than baseline level [17]. MBL deficiency is generally defined by plasmatic protein levels below 500 ng/mL or by an MBL function lower than 0.2 U/*μ*L C4 deposition [18]. The detection of pulmonary MBL concentration is quite difficult. Some authors have reported bronchoalveolar lavage (BAL) levels ranging between 20 and 80 ng/mL, but these results were not corrected for dilution factors (i.e., urea or other lung proteins with known lung concentration) that could explain the large distance from the minimum concentration needed to activate complement proteins (300-400 ng/mL) [19]. Plasmatic levels ranging between 500 and 1000 ng/mL are generally detected in heterozygous patients (A/O genotype); instead homozygous variant MBL2 alleles usually present very low concentrations (<50 ng/mL). Similarly the haplotypes that include mutations in promoter regions are associated with significant reduction of MBL protein production and activity. However, even though the degree of MBL deficiency is strictly dependent upon patients' genotypes, in some cases low MBL plasmatic levels have been also associated with wild-type genes [13]. This gene was already present in early invertebrates more than fifty million years ago and has been highly conserved throughout animal and human evolution. This would suggest that the correct function of MBL protein is crucial for the survival of living animal species. It is of interest to note that there is geographic distribution of different alleles: the B variant is predominant in Eurasian populations, the C variant mainly among Asians and America Indians, and the D haplotype seems to be frequently expressed in Caucasian region. This particular distribution might be linked to the initial human migrations out of Africa and induced by some specific advantages due to MBL deficiency. For example, high MBL production has been observed to be associated with higher incidence of preterm births; instead moderately low levels could protect the organism from mycobacteria systemic infection and from the complement induced inflammatory-mediated damage of some diseases (i.e., meningococcemia and rheumatoid arthritis) [20, 21]. Additionally sporadic reports have not found a clear association between MBL deficiency and increased rate of infectious episodes [22–24]. The wide range of clinical effects linked to MBL haplotypes has also been attributed to the role of associated mutations in other genes encoding proteins with similar functions (i.e., L-ficolin, MASP2, and surfactant proteins) [25, 26]. However, to date, there are few data upon the clinical role of these combined deficiencies.

## 3. MBL and Severe Infections

The prevalence of mutations in one or both MBL2 gene alleles is relevant, ranging between 30% and 40% in analyzed populations [13, 27]. During the last twenty years an increasing body of evidence has indicated that MBL deficit, due to specific haplotypes, generally increases frequency and severity of infectious episodes [28, 29] (Table 1). However the structure of our immune system is redundant, and this may explain why in many cases polymorphisms of MBL2 gene were not observed to influence susceptibility to infections [24]. The role of this lectin is particularly relevant when adaptive immune system is immature or compromised [30]. In a case-control study upon 47 infants, lower MBL cord blood concentrations were associated with a higher incidence of Gram-negative sepsis (*P* = 0.036) [31], and an observational cohort study upon 100 pediatric ICU patients identified MBL2 gene exon 1 polymorphisms as a main determinant of progression from sepsis to septic shock [32]. Additionally, the incidence and outcome of severe infections appear to be influenced by the levels and activity of mannose-binding lectin. In a cohort of leukemic patients undergoing chemotherapy, severe infections (bacteremia, pneumonia or both) occurred more frequently in those individuals with lower MBL concentrations (*P* < 0.001) [33]. In an ethnically homogeneous English population, homozygotes for MBL codon variant alleles showed a significantly higher risk of invasive infections due to *Streptococcus pneumoniae*, “the captain of men of death” [34]. Similarly allelic variants of this gene seem to be associated with increased susceptibility to meningococcal disease [35]. Among respiratory tract infections, independently from the causal pathogen, MBL insufficiency has been observed to predispose to higher severity and poor outcome [36]. Even though *Legionella spp.* act as an intracellular pathogen, MBL function was lower in infected cases during an Australian *Legionnaires' disease *outbreak [37]. Increased susceptibility and worse outcome in 212 Caucasian patients with acute respiratory distress syndrome (ARDS) were also observed in presence of MBL2 gene polymorphisms [38]. Regarding viruses, in Chinese population, the presence of MBL2 gene B variant was associated with increased risk of *Coronavirus *infection [39, 40]; instead normal MBL function seems to worsen pandemic H1N1 and avian H9N2 infections by potentially upregulating inflammatory response [41]. 

Furthermore, in a recent large retrospective study involving 102 donor-recipient orthotopic liver transplantation pairs, patients who received MBL-deficient livers showed a threefold increased risk of clinically significant infections including *Cytomegalovirus*-related diseases [42].

Few authors have studied the role of MBL in severe fungal infections. Polymorphisms of this gene were observed in seven of ten white patients with chronic necrotizing aspergillosis compared with 25% of controls [43]. In addition variations of MBL plasmatic levels seem to correlate with the occurrence of invasive candidiasis [44]. 

MBL genetic, plasmatic, and functional profiles were investigated in numerous clinical settings obtaining different results. The critically ill patient, affected by severe infections with severe sepsis and septic shock, might be a field of particular interest for a better knowledge of their clinical relevance and the possible development of novel therapeutic strategies.

## 4. MBL and Severe Sepsis/Septic Shock

Mannose-binding lectin is not only part of the innate recognition system of invasive pathogens but effectively modulates the cytokines' production by macrophages during phagocytosis. This effect, upon interleukin (IL)-6, IL-1*β*, and tumor necrosis factor-**α**, was clearly shown in an “*ex vivo*” model of immediate immunity response to *Neisseria Meningitidis* infection [45]. MBL deficiency may be associated with unbalanced proinflammatory responses to infective and noninfective triggers. In a cohort of critically ill pediatric patients, *Fidler and coworkers *observed that MBL levels less than 1000 ng/mL, consistent with MBL-2 gene exon 1 polymorphisms, significantly increased the risk of developing systemic inflammatory response syndrome (SIRS) and progression to severe sepsis/septic shock [32]. Additionally in patients with SIRS, MBL insufficiency degree was observed to correlate with severity of systemic infection, according to the genetic profile [46]. The association between the deficiency of this protein and worse outcome during severe systemic infections (i.e. evolution to refractory septic shock) may be also related to the significant interaction between complement activation, inflammatory cytokines' “storm”, and coagulation cascade. The influence of complement activation upon septic shock development was largely investigated. Many studies have shown how the classical and alternative pathways are activated during septic shock and are involved in mechanisms aimed to clear endotoxin. This role has been more recently studied also for lectin complement activation due to MBL [47, 48]. Disseminated intravascular coagulation (DIC) may worsen the course of septic shock but the occurrence of this severe complication is unpredictable. However recent data suggest that MBL deficit may be a significant risk factor for the early development of DIC and organ failure during severe infections [9]. Conversely, excessive MBL expression might be harmful, since this molecule may contribute to the pathogenesis of inflammatory induced vascular damage and organ failure, as observed in patients undergoing solid organ transplantation [49]. Hence MBL, due to its pivotal role in the crosstalking among complement activation, coagulation, and systemic inflammation, may represent a key point for the understanding of the development of systemic severe infections, as interestingly investigated in animal models and clinical studies involving patients with severe sepsis/septic shock. 

### 4.1. Animal Models

Even though many differences between animal models and humans limit the “translationalability” of preclinical data, several mouse experiments support the role of MBL deficiency in severe infections, especially after bacteria inoculation. Two functional MBL genes exist in the mouse, and the generation of double knockout gene-deficient mice has increased the investigations in this field.

After inoculation of 5 × 10^7^ CFU *Staphylococcus Aureus*, MBL-null mice showed at 48 hours 100% mortality compared with wild-type (WT) mice which survived in a percentage of 55%. Additionally, pretreatment of MBL-null mice with rhMBL increased their survival rate of about 50% [50]. In another model of MBL and/or MASP 1/3 deficient mice *Takahashi and coworkers* observed that this deficiency was associated with early occurrence of DIC and liver injury after *S. aureus* inoculation, suggesting the role of this protein in the development of organ failure and systemic coagulation activation during severe infections [9].

Another study demonstrated that MBL is able to strongly bind to O-antigen region of LPS, contributing to mice platelets activation and rapid occurrence of septic shock [48].

Susceptibility of MBL null mice to *Pseudomonas aeruginosa *postburn infection was also investigated [51]. All MBL-null mice, after burn and bacterial inoculation, early developed septic shock and died; instead the majority of WT animals (two-thirds) survived. These observations underline the relevance of innate immunity and mannose-binding lectin in the susceptibility and outcome of severe bacterial infections occurring in this population.

Regarding fungal diseases, the protective role of this lectin was also observed in murine models of invasive pulmonary aspergillosis after *ex vivo* MBL administration [52].

However lectin pathway activation does not only depend on MBL function. Some authors have observed how deficient mice models, without the capability to activate MBL-independent lectin cascade (i.e., ficolins and other collectins), are more susceptible to develop severe systemic pneumococcal infections [53].

Although most of literature evidence obtained by animal studies supports the importance of MBL in the acquisition and outcome of severe infections, these observations, due to unresolved several limits of animal studies, may not be considered conclusive and strongly need clinical human studies to definitely identify its clinical relevance. 

### 4.2. Human Pediatric and Adult Studies

The MBL key role as part of innate immunity is the reason why haplotypes associated with its deficiency mainly influence infectious episodes involving neonates and children or immunosuppressed adults. In a population-based prospective study performed in Greenland upon almost 300 Eskimo children, both heterozygous and homozygous subjects, aged 6 to 17 months, for variant alleles presented a twofold increased risk of acute respiratory infections, including pneumonia [30]. Additionally, *Capoluongo and colleagues*, analyzing 75 preterm newborns, identified two MBL2 gene variants as independent risk factors associated with unfavorable outcome, including higher bronchopulmonary dysplasia prevalence [54]. Turkish authors have investigated the possible relationship between cord blood MBL levels and neonatal sepsis. The results indicated that lower MBL levels during fetal inflammatory response syndrome (FIRS) were associated with higher risk of sepsis development independently from gestational age and birth weight [55]. Another prospective study conducted on 62 neonates (27 of them were preterm) showed how lowest MBL levels were detected in infants with septic shock, especially in case of fatal outcome (*P* < 0.05). Relevant sensitivity, specificity, positive, and negative predictive values for detecting sepsis episodes were also documented [56].

In a recent Swiss investigation, MBL levels were detected in cord blood of 141 newborns. Forty-seven developed sepsis (28% within the first 72 hours of life) and 13% required catecholamines because of septic shock. After excluding those infants who underwent surgery, low MBL concentrations resulted independently being associated with increased risk of early-onset Gram-negative sepsis [31].

In pediatric oncological patients, MBL deficiency was associated with susceptibility, poor outcome, and duration of febrile neutropenic episodes [57]. In a prospective study MBL deficit was observed to increase the severity of disease during pediatric ICU admission after febrile neutropenia [58]. Additionally also MBL-related proteins deficit was investigated in this setting. In a cohort of 94 children treated with chemotherapy for cancer, MASP-2 deficit (<200 ng/mL) significantly increased the risk of febrile neutropenia and bacteraemia development and prolonged cumulative duration of hospitalization and antimicrobial treatment [59].

The importance of MBL function during the first months of life, when the efficacy of innate immunity is crucial, has induced some authors to propose its dosing as part of a biomarkers panel for the early detection of severe neonatal infections in low-resource settings [60].

Impaired innate immune mechanisms may also increase the risk of nosocomial infections in critically ill patients as observed by *Sutherland and colleagues*. In a genetic association study, the authors identified the relationship between SNP in CD 14, MBL and Toll-like receptor-2 with increased prevalence of positive cultures and sepsis [61]. In a cohort of 195 adult septic patients, MBL deficiency resulted also independently being associated with higher sequential organ failure assessment (SOFA) score at day 3, suggesting its role as a risk factor for the development of severe sepsis and septic shock [62]. Additionally in a multicenter prospective study involving eight adults ICUs in U. K., the association between MBL-2 exon and promoter polymorphisms with the outcome of 174 patients affected by severe sepsis and septic shock was studied [63]. Compared with healthy subjects, MBL deficient patients were at increased risk of sepsis, with a significant higher mortality rate in presence of levels below 1000 ng/mL (47.2% versus 22.2%, *P* = 0.05). 

During severe sepsis and septic shock, the increase of MBL plasmatic levels, as acute phase response molecule, may be different. In a report of 128 adult critically ill patients, *Dean and colleagues* observed that regardless of MBL-2 genotype those patients who were MBL deficient at study entry were not able to reach normal plasmatic levels during severe sepsis and septic shock [17]. Furthermore, a well-conducted prospective study, performed in Denmark, investigated the MBL genetic and plasmatic profile in a population of 272 critically ill ICU patients with documented SIRS [46]. Among enrolled patients 172 met the criteria for severe sepsis and 70 for septic shock. Compared with noninfectious SIRS, these patients shared the carriage of MBL variants alleles and low serum levels according to the severity of disease (*P* = 0.03). 

Another recent Korean study in ICU patients investigated whether MBL2 gene polymorphisms and serum levels might influence severity and prognosis of sepsis [64]. 

The authors compared 26 septic patients with 398 healthy controls, analyzing three SNP and dosing MBL serum levels on day one. Among sepsis group, homozygosis for the polymorphism at codon 54 (A/A) resulted in a significant risk factor for severe sepsis development (*P* = 0.001). MBL serum levels ≥ 1.3 mcg/mL were associated with a lower 28-day mortality rate in the septic shock group (*P* = 0.02).

The role of MBL deficiency in critically ill patients with severe pneumonia, a still leading cause of death due to an infectious disease, has been investigated by many authors. In a large case-control study, 848 patients affected by community-acquired pneumonia (CAP) were compared with 1447 healthy control subjects and 519 patients without relevant infectious diseases. MBL2 and MASP2 haplotypes were equally distributed among those subjects. In the multivariate analysis, MBL deficiency was associated with poor outcome measures (i.e., severe sepsis, acute respiratory failure, multiorgan dysfunction syndrome, and death) [36]. 

*Eisen and colleagues* have reanalyzed data from six studies involving 675 patients affected by severe infections [18]. First, the authors defined a MBL cutoff value of 0.5 mcg/mL as a reliable predictor of low producing status (negative predictive value 98%). They confirmed that MBL deficiency significantly increased the risk of death due to severe infection, also in ICU setting, especially when *Streptococcus Pneumoniae* was the invasive causative agent (odds ratio 5.6, 95% confidence interval, 1.27–24.3). The association between MBL deficiency and *S. pneumoniae* invasive infection outcome has been recently investigated in a Spanish prospective cohort study [65]. During the study period 117 patients with invasive pneumococcal infection were enrolled: the rate of allelic variants was 32%. SNP MBL2 (AO/OO) and septic shock were the factors independently associated with in-hospital mortality. Otherwise early adequate antibiotic dose ≤ 4 hours resulted in a significant protective determinant. 

MBL deficiency role was also studied in some other systemic infections due to specific organisms. *Resman and colleagues* recently described the case of a necrotizing myositis and septic shock due to *Haemophilus Influenzae* in a patient where IgG3 and MBL deficiency were diagnosed [66]. In a well-conducted prospective study, the correlation between MBL2 gene polymorphisms and the outcome of *Escherichia coli* pyelonephritis was investigated [67]. Although no association was found with the incidence of *E. coli* infections and the presence of bacteremia, those patients who shared low-expression MBL2 genotypes showed a significant higher risk of septic shock development (odd ratio: 9.1, 95% confidence interval: 1.23–65.9; *P* = 0.03). Finally, in nonbacterial severe systemic infections, invasive candidiasis (IC), especially candidemia, still remains a leading cause of death due to infections in critically ill patients. Serum MBL levels were measured in 68 patients with proven IC, 82 hospitalized not infected patients, and 70 healthy subjects [44]. Even though MBL concentration was significantly higher in IC patients than controls, the authors identified a marked decrease in its plasmatic levels during the first days of infection in association with mannans increase. These observations, although limited, suggest a crucial role of MBL also in the early phase of candidiasis.

### 4.3. MBL Replacement Therapy

MBL substitution therapy in patients with recognized lectin deficit has been proposed. Apart from genetic analyses, antigenic measurement is widely diffused as diagnostic test. Even though MBL serum levels < 500 ng/mL or MBL activity < 200 U/mL may be considered a significant deficiency, there are not standard guidelines aimed to define which patient categories need to be tested (i.e., in presence of severe recurrent respiratory infections or acquired immunesuppression). Recombinant human MBL use, to supplement MBL deficiency status, has been investigated in animal and phase I/II human studies [68, 69]. Although its clinical efficacy has not been clearly established, still now no adverse effects were observed. Sixty-five MBL infusions were given to 12 MBL deficient chemotherapy-induced neutropenic children. The observed postadministration level was 1.06 mcg/mL (range: 0.66–2.05) which may be considered protective [70]. A similar pharmacokinetic profile was observed in 20 healthy MBL-deficient volunteers and two patients with *Staphylococcus Aureus* septicemia [71]. However, beyond these preliminary observations, MBL replacement needs to be further investigated in deficient patients affected by acute severe infections, especially in presence of multiple-level immune system impairment.

## 5. Conclusions

An increasing body of data support the role of MBL as central player of innate immunity. Several gene polymorphisms have been identified in association with decreased serum levels and activity. Many authors have showed the association of this molecule deficit with recurrent severe infections, particularly involving the respiratory tract and encapsulated bacteria. Additionally growing evidence suggests its importance during systemic severe infections as severe sepsis and septic shock. This correlation might derive from the cross-talking among complement system, coagulation patterns, and proinflammatory cytokines. Even though many patients with systemic infections, who present MBL serum levels below the functional threshold, are at higher risk to develop severe complications and poor outcomes (i.e., septic shock, multiple organ failure), in some cases low levels have appeared to be protective, probably reducing the inflammatory cytokines' storm. Moreover not all published studies have identified a clear association between deficiency and increased risk of infections. Replacement therapy with recombinant human protein during severe sepsis and septic shock affecting deficient patients has been proposed but it still remains an experimental treatment. Hence, until new promising and robust data will be available, the strict adherence to current standard recommendations still remains the mainstay of severe sepsis/septic shock management. 

## Figures and Tables

**Table 1:** MBL deficiency and susceptibility to diseases (human and animal studies).

Bacterial infections	Viral infections	Parasitic infections	Fungal infections	Miscellaneous
(i) *Streptococcus pneumoniae *	(i) CMV	(i) *Plasmodium falciparum *	(i) *Candida spp *	(i) Recurrent respiratory infections
(ii) *Neisseria meningitidis *	(ii) Coronavirus	(ii) *Cryptosporidium parvum *	(ii) *Aspergillus spp. *	(ii) Chemotherapy-induced febrile neutropenia
(iii) *Staphylococcus aureus *	(iii) HIV			(iii) Systemic lupus erythematosus
(iv) *Pseudomonas aeruginosa *	(iv) HCV			(iv) Chronic renal failure
(v) *Escherichia coli *	(v) HBV			(v) Cystic fibrosis
(vi) *Legionella spp. *	(vi) HSV			
